# Optimizing Bipolar Reset Waveform to Improve Grayscale Stability in Active Matrix Electrowetting Displays

**DOI:** 10.3390/mi15101247

**Published:** 2024-10-11

**Authors:** Taiyuan Zhang, Li Wang, Linwei Liu, Wei Li, Shipeng Wu, Jianyang Guo, Guofu Zhou

**Affiliations:** 1 Guangdong Provincial Key Laboratory of Optical Information Materials and Technology & Institute of Electronic Paper Displays, South China Academy of Advanced Optoelectronics, South China Normal University, Guangzhou 510006, China; ztyuan668@163.com (T.Z.); yoeksome@sina.com (L.L.); wei.li@guohua-oet.com (W.L.); wushipeng@m.scnu.edu.cn (S.W.); 2 Administrative and Research Office, South China Normal University, Guangzhou 510006, China; guojy@scnu.edu.cn; 3 School of Information Engineering, Zhongshan Polytechnic, Zhongshan 528400, China; wangli@zspt.edu.cn; 4 Shenzhen Guohua Optoelectronics Technology Co., Ltd., Shenzhen 518110, China

**Keywords:** electrowetting display, oil backflow, driving waveform, active matrix, grayscale stability

## Abstract

The electrowetting display (EWD) device is a new type of electrowetting-on-dielectric (EWOD) equipment that can achieve a paper-like display effect under the control of an electric field. In this microfluidic system, the stability of grayscale can be affected by various factors, such as the physicochemical properties of the materials, the device structure, and electric field distribution. To improve the grayscale stability of active matrix electrowetting displays (AM-EWDs), the impact of different polarities of driving voltage on oil backflow was investigated in this study. Based on the driving principles of AM-EWD, an optimized inter-frame bipolar reset driving waveform was designed to overcome oil backflow. The proposed driving waveform maintained the stability of the oil state by periodically and rapidly releasing trapped charges in the dielectric layer through a reverse driving voltage. Additionally, the effect of feed-through voltage on pixel driving voltage was eliminated by compensating for the driving voltage on a common electrode. Finally, the performance of the designed driving waveform was evaluated with a 6-inch AM-EWD driving platform. Compared to the conventional unipolar reset driving waveform, the backflow speed decreased by 2.70 a.u./s. The standard deviation of the display luminance was also reduced by 11.24 a.u. Experimental results indicated that both the oil backflow speed and the fluctuation range of luminance were effectively suppressed by the proposed driving waveform.

## 1. Introduction

Electrowetting is a phenomenon which changes the wettability of a liquid on a solid surface by applying a voltage between electrodes [1,2,3,4]. By changing the applied voltage, the contact angle of the liquid can be adjusted, thereby controlling the shape and position of the liquid on the solid surface [5]. Electrowetting has a wide range of applications in various fields, including microfluidics [6,7], energy harvesting [8], nanogenerators [9], optical devices [10,11], and display technologies [12,13]. Among them, electrowetting display (EWD) is a reflective display technology based on electrowetting [14]. In the field of EWD technology, an external electric field is employed to manipulate the shape of liquid within pixels. Under the modulation effect of liquid on light, images can be displayed on the EWD device. Unlike conventional liquid crystal display (LCD) technology, EWD does not require a backlight. Therefore, EWDs have extremely low power consumption [15]. Additionally, the shape of the liquid in pixels can be changed within milliseconds, resulting in a fast response speed [16].

Despite the advantages of EWDs, there are still some issues in practical applications [17,18,19]. The oil backflow phenomenon is one of the most influential issues on display performance [20]. The adverse effects of oil backflow on the performance of EWDs include a decrease in contrast, grayscale distortion, and instability of image display [21]. The occurrence of oil backflow is closely related to the process flow, material system, and the physicochemical properties of the dielectric layer. Studies have shown that charge trapping is an important cause of oil backflow [22,23,24]. Verheijen proposed a charge trapping model in reversible electrowetting and provided methods for measuring the density of trapped charges [25,26], laying the experimental and theoretical foundations for subsequent studies on charge trapping phenomena. The oil could not maintain a stable state because of the local electric field, which was generated by trapped charges [27]. As a result, pixels of the EWD panel demonstrated unstable grayscales when the charge trapping phenomenon occurred [28]. Furthermore, defects and impurities in the dielectric layer exacerbated the degree of oil backflow by accelerating the trapping of charges. To suppress oil backflow and improve the stability of EWDs, researchers have conducted extensive studies on pixel structure, material systems, process flow, and driving waveforms [29]. Driving waveforms have attracted significant attention due to their ability to achieve notable effects in a short period. The basic strategy for suppressing oil backflow was to release the trapped charges in the dielectric layer [30]. A well-designed driving waveform for EWDs can release the charges trapped by the dielectric layer in a timely manner rather than accelerating the accumulation process of the trapped charges [31]. For passive matrix electrowetting displays (PM-EWDs), a periodic reset signal was introduced in direct-current (DC) driving to suppress oil backflow [32]. Similar driving strategies were also adopted in a study of other driving waveforms, which were used to suppress oil backflow. Compared to PM-EWDs, active matrix electrowetting displays (AM-EWDs) have more complex pixel structures and driving circuits. Therefore, the driving waveforms designed for PM-EWDs cannot be directly applied to AM-EWDs. In the driving of AM-EWDs, a method using asymmetric alternating-current (AC) driving waveform was proposed to achieve high performance EWDs by the suppression of charge trapping [33]. In addition, a separated reset waveform with a charge release phase and an oil recovery phase was proposed to suppress oil backflow [34]. More recently, an asymmetric intermediate frequency AC driving model was proposed for improving the quality of image display [35]. In the driving circuit of AM-EWDs pixels, the feed-through voltage generated by electromagnetic coupling or capacitive coupling between different signal lines caused the driving voltage of pixel electrodes to deviate from the design value. However, the effect of feed-through voltage on pixel driving voltage was not considered in these studies of driving waveforms. These strategies would introduce new unstable factors to the display of images on AM-EWDs.

To suppress oil backflow in AM-EWDs and improve grayscale stability, an optimized bipolar reset driving waveform is proposed in this paper. Firstly, the effect of different polarities of driving voltage on oil backflow in AM-EWDs was studied. Then, a driving system for a 6-inch AM-EWD panel was designed for evaluating the performance of the driving waveform. Finally, a method for eliminating the influence of feed-through voltage on the driving voltage of pixels was proposed based on the driving principles of AM-EWDs. The proposed driving waveform improves the display stability of EWDs by suppressing oil backflow. This is of great significance for improving the accuracy and number of grayscale levels in AM-EWDs.

## 2. Oil Backflow Phenomenon in AM-EWDs

**Figure 1:**
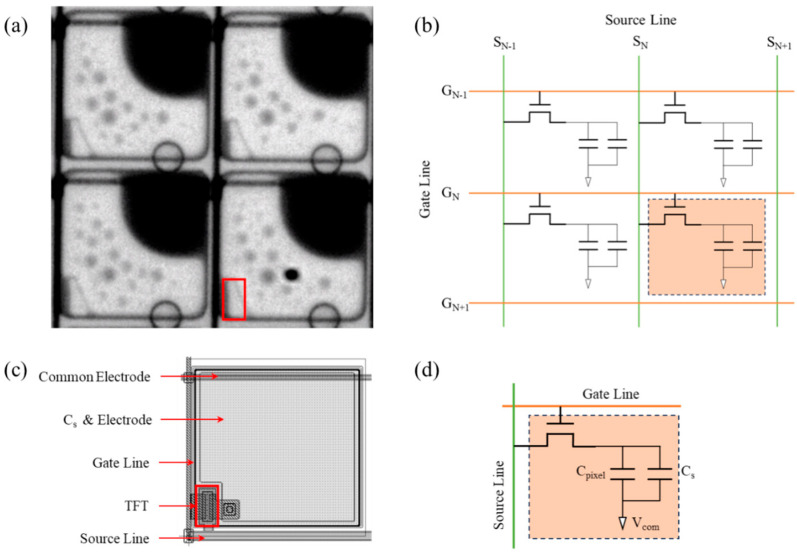
Pixels of AM-EWD and its equivalent circuits. (**a**) Black EWD pixels in an active state. The TFT structure is located at the position of the red rectangle in the pixel. (**b**) Equivalent circuit of TFT panel (partial). (**c**) Pixel structure diagram. (**d**) The equivalent circuit of a TFT with Cs on common structure.

The fundamental principle of EWD technology involves the manipulation of electric fields to control the contact angle between the colored oil and the surface of the dielectric layer. Similar to LCD technology, the driving methods for EWDs can also be categorized into two types: PM driving and AM driving. In PM driving, a high-speed point-by-point scanning manner was used to active pixels of the display panel. PM driving can offer a better price advantage because of its simple structure and mature technology. However, crosstalk phenomena are more likely to manifest when the PM-based screen resolution is high. Compared to PM driving, AM driving can achieve a higher brightness at a lower power consumption. In addition, AM driving is also beneficial for improving the integration of devices. The pixel structure and equivalent circuit of the AM-EWD are depicted in Figure 1. In AM driving, a thin-film transistor (TFT) was incorporated at each pixel to function as a switch, as depicted in Figure 1a. In the presence of an electric field, the black oil shrank to a corner of the pixel. The equivalent circuit diagram of the TFT matrix is illustrated in Figure 1b. The output of TFT was determined by the signals on the corresponding source line and gate line. Each EWD pixel in Figure 1c can be regarded as a capacitive load $C_{pixel}$ in the TFT driving circuit, as illustrated in Figure 1d. $C_{s}$ represents the storage capacitor in the pixel driving circuit. The driving voltage for each pixel can be accurately regulated by controlling the conduction state of the TFT. This driving technique is distinguished by its high refresh rates and exceptional image quality. Hence, AM driving is considered suitable for EWD applications that require high resolution and large size. In AM-EWD, the magnitude of the driving voltage applied to the indium tin oxide (ITO) electrode is dictated by the conduction state of the TFT. Fluctuations in the driving voltage can lead to variations of the contact angle of the colored oil. The adjustment of the aperture ratio of the pixel is achieved through variations in the contact angle of the colored oil, thus enabling the display of grayscales.

**Figure 2:**
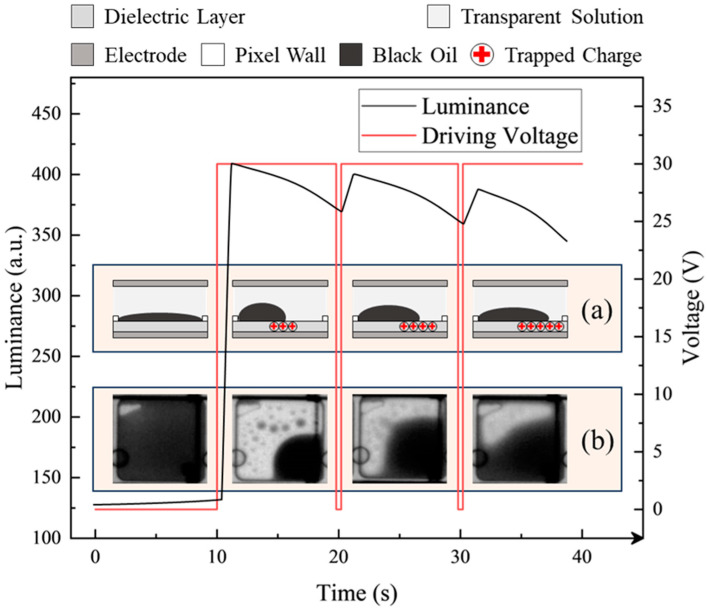
The effect of periodic reset signals on EWDs. (**a**) Variation in the quantity of trapped charges within the dielectric layer. (**b**) Oil states of the pixel.

In addition to the pixel circuit structure, the response of oil to an electric field is also a fundamental aspect that must be taken into account when designing driving waveforms. The previous experimental results indicated that prolonged direct-current (DC) driving of pixels can lead to instability of the contracted oil [32]. As the driving time increased, the aperture ratio of pixels decreased, causing a phenomenon known as oil backflow. This phenomenon resulted in a sustained decrease in the luminance of EWDs, impacting the overall display performance. The behavior of EWD pixels under unipolar reset driving conditions is illustrated in Figure 2. The fundamental structure of an EWD pixel typically consists of a driving electrode, a dielectric layer, a transparent liquid, colored oil, and a pixel wall. A schematic representation of the stack structure of a pixel is provided in Figure 2a. The accumulation of charge on the surface of the dielectric layer when a voltage was applied to the pixel electrode was noted, as depicted in Figure 2a. The accumulated charges heightened the polarization of the dielectric layer surface under the influence of electric field forces. This process changed the intensity of the pixel electric field and disturbed the equilibrium of the three-phase contact line, ultimately resulting in oil backflow. Pixel state during oil backflow is depicted in Figure 2b.

Figure 2 depicts the complete spreading process of oil within a pixel in the absence of voltage application during the first 15 s. Subsequently, a 30 V DC driving featuring periodic reset waveform was applied to the pixel within the following 30 s. During this period, the EWD pixel exhibited the phenomenon of oil backflow. The luminance showed a slight recovery under the influence of the reset waveform. It was indicated that oil backflow can be partially suppressed by the reset waveform [31]. However, the suppression effect of the unipolar reset waveform on oil backflow was limited. Simultaneously, it can induce screen flickering as a result of excessive fluctuations in luminance. In fact, the oil backflow phenomenon can be divided into two categories: reversible backflow and irreversible backflow. For reversible backflow, the oil could revert back to its original state when the same driving voltage was reapplied. During the driving process, some polar molecules within the liquid medium were dissociated under the influence of an electric field. The formed charges were migrated towards the dielectric layer under the influence of electric field forces and accumulated on its surface. The substantial accumulation of charges on the dielectric layer surface intensified the local electric field, enhancing the polarization effect of the dielectric layer. The accumulated charges were discharged after the removal of the driving voltage. Therefore, the oil within the pixel could return to its original state. Nevertheless, the dielectric layer might contain some specific defects or discontinuities due to factors of process or material. When the oil was contracted, water molecules or impurities in the solution made contact with electrodes on the substrate through these defects or gaps, which resulted in direct conduction between the upper and lower substrates through the water layer, thereby diminishing the role of the dielectric layer. In addition, the penetrated water molecules or impurities may participate in electrochemical reactions with the ITO electrode on substrates, which could result in alterations to the properties of the dielectric layer. The aforementioned processes were irreversible, thereby preventing the pixel from reverting to its original state after the removal of the electric field.

## 3. Experimental Methods

### 3.1. Experimental Platform

**Figure 3:**
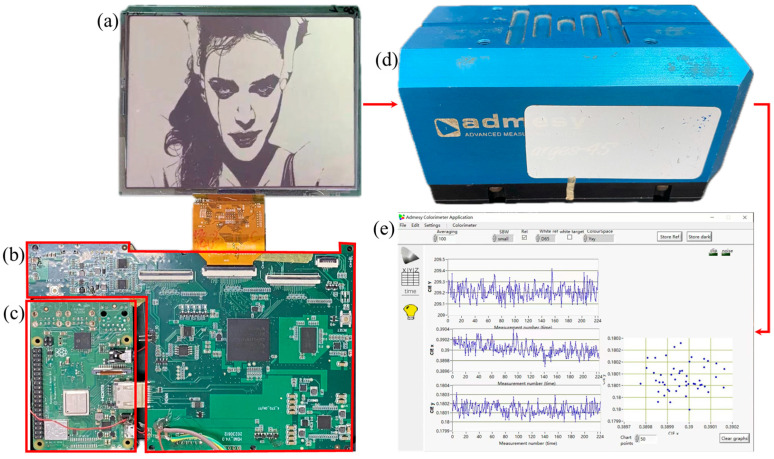
The experimental system. (**a**) An EWD panel. (**b**) The driving board for EWDs. (**c**) The control board. (**d**) The colorimeter. (**e**) Luminance data acquisition software.

It was necessary to evaluate the performance of the proposed driving waveform by the display effect of the AM-EWDs panel. Therefore, an experimental platform was built to evaluate the performance of the designed reset driving waveform in maintaining the luminance of EWDs. The experimental system comprised a control board, a driver board, an EWD panel, and an Arges-45° colorimeter manufactured by Admesy, as shown in Figure 3.

The control board was served by a development board (Raspberry Pi 3 Model B+) equipped with the Ubuntu operating system. It was responsible for reading video or image files from the micro secure digital (SD) card and transmitting them to the driver board through the high-definition multimedia interface (HDMI). The driver board carried a field programmable gate array (FPAG) device GW2A-LV18PG484C8/I7, which was manufactured by Gowin Semiconductor Corp. The chip has rich digital signal processing (DSP) resources and rich block static random-access memory (BSRAM) resources. Therefore, it could be used for high-speed communication such as low-voltage differential signaling (LVDS) and HDMI transceiver. Once the video data stream was received by the driver board, the FPGA chip was responsible for parsing the pixel data of each image frame from the data stream. Based on the obtained pixel data, the FPGA chip determined the voltage value for the corresponding pixel electrode according to the preset image processing algorithm and driving waveforms. A 6-inch display panel with black oil in the pixels was used in the experiments. The fabrication of an EWD panel required a series of processes, including photolithography, inkjet printing, adhesive encapsulation, and chip bonding. The detailed parameters of the panel, which was developed by South China Normal University, are presented in Table 1. To detect changes of the EWD luminance in real time, the colorimeter was placed above the viewing area on the EWD screen. The host computer software that matched with the colorimeter would collect and save the luminance data obtained by the colorimeter through a universal serial bus (USB) for further analysis and processing. All designed experiments were performed at room temperature.

### 3.2. Oil Backflow under Different Voltage Polarity

**Figure 4:**
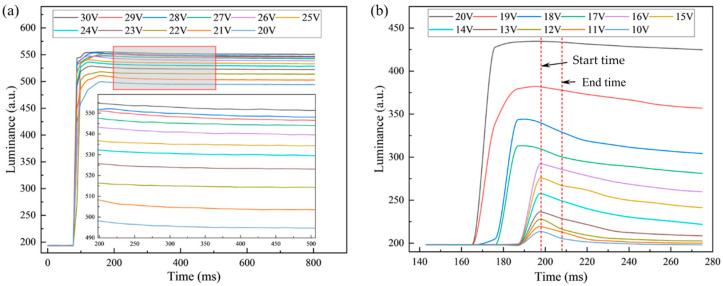
The influence of driving voltage polarity on luminance curves. (**a**) Positive driving. (**b**) Negative driving.

For addressing the phenomenon of oil backflow in EWDs, periodic reset signals were considered to be one of the most effective strategies to suppress oil backflow [23]. A common reset method was to set the upper and lower electrodes to the same voltage, attempting to release the trapped charges in the dielectric layer by applying a zero voltage. However, experimental results revealed that there were significant differences in the speed of oil backflow under driving voltages of different amplitudes and polarities. The oil backflow curves for positive driving and negative driving at different voltage amplitudes are shown in Figure 4. The graph illustrates the variations in luminance of the EWD panel before and after the application of voltage, as well as during the oil backflow process.

From the oil backflow curves of positive driving in Figure 4a, it was found that the luminance curve trends under different driving voltages were basically consistent when the luminance reached the maximum value. In addition, the luminance difference between adjacent voltages decreased as the amplitude of the driving voltage increased. The fundamental trend of oil backflow curves driven by negative voltage in Figure 4b were consistent with those driven by positive voltage. However, the luminance variation between adjacent driving voltages increased with the increase of voltage amplitude. It was worth noting that a higher driving voltage amplitude was required for negative driving to match the luminance achieved with positive voltage driving. Comparing Figure 4a and Figure 4b, it could be seen that the oil backflow speed under negative driving was much higher than that under positive driving, especially when the luminance had just reached the maximum value. This was due to the fact that the charge trapping effect occurred continuously throughout the entire driving process; the dielectric layer under negative driving exhibited enhanced charge trapping capability and speed. This characteristic indicated that a short-term negative driving pulse may be more effective in suppressing the oil backflow phenomenon.

The direct consequence of the oil backflow phenomenon was a continuous decrease in luminance of the EWD panel. Therefore, the average rate of luminance decrease over a period of time was used as an indicator to the speed of oil backflow. For the positive driving, a time range from 200 ms to 500 ms was selected. For the negative driving, the selected time period started at 198 ms and ended at 208 ms. During the initial power-on of the device, the movement of oil and the charge trapping occurred simultaneously. Thus, the process of charge trapping could not be fully represented by the variation of luminance at that time. The spreading process was closely related to charge trapping only after the oil had shrunk to the maximum extent. Therefore, the luminance variations within these two designated time periods were selected to characterize the rate of oil backflow.

**Figure 5:**
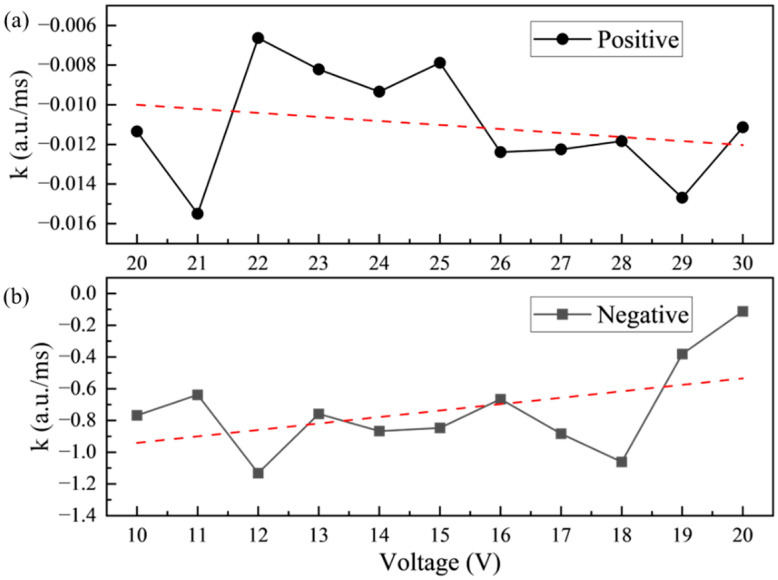
The relationship between the rate of luminance variations and the driving voltage. (**a**) Luminance reduction rate under positive voltage. (**b**) Luminance reduction rate under negative voltage.

To observe the difference, we plotted the relationship curves of oil backflow speed versus driving voltage amplitude under positive and negative driving conditions, as illustrated in Figure 5. It can be seen that there was a significant difference in the oil backflow speed of EWD under two polarity driving voltages. The luminance difference indicated that the oil backflow speed exhibited voltage polarity dependence. Compared with negative driving, the oil backflow speed under positive driving was lower and had a smaller fluctuation range. The trend of oil backflow speed is indicated by the red dashed line in the figure. In the positive driving, the oil backflow speed under different driving voltages could be approximated as a constant value. Unlike the positive driving, the oil backflow rate decreased with increasing voltage amplitude in the negative driving. This phenomenon indicated that charges were more easily trapped by the dielectric layer when the negative voltage was applied.

By systematically studying the influence mechanism of driving voltage polarity on charge trapping speed, an important basis was provided for optimizing the driving mode and material selection of EWDs. When designing a driving circuit, it was necessary to fully consider the different trapped charge amounts of positive and negative polarity voltages. The negative impact of charge trapping on device performance was mitigated by choosing a reasonable driving waveform.

### 3.3. Design of Driving Waveform

**Figure 6:**
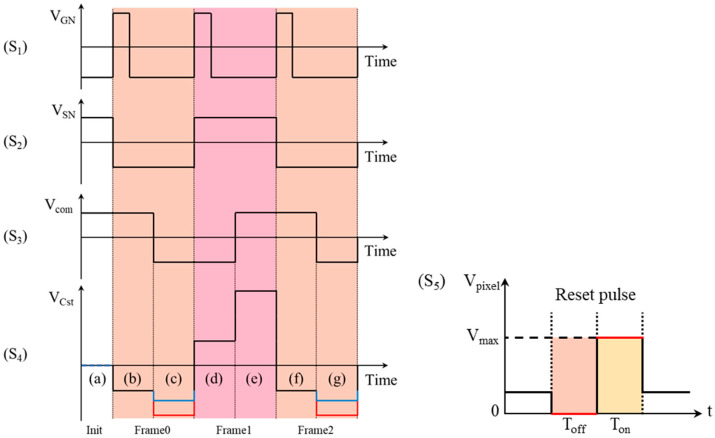
Schematic diagram of conventional reset driving waveform. (S_1_) Gate signal. (S_2_) Source signal. (S_3_) Reverse signal on the common electrode. (S_4_) The potential difference between the storage capacitor and the source electrode of the TFT. (S_5_) Schematic diagram of conventional unipolar reset driving waveform. (a) Initial state. The two different stages of the first frame are (b) and (c). The two different stages of the second frame are (d) and (e). Stages (f) and (g) are the same as in the first frame.

Previous studies showed that flipping the voltage polarity was an effective method to eliminate the charges trapped by the dielectric layer in electrowetting-on-dielectric (EWOD) devices [36]. During the driving process of EWDs, the driving waveform with unchanged voltage polarity was called the unipolar driving waveform. The driving waveform composed of positive and negative voltages was called the bipolar driving waveform. In the case of AM-EWDs, inserting a reset driving waveform between driving frames would occupy the frame time. The conventional unipolar reset driving waveform is shown in Figure 6 (S_5_) [30]. It was typically comprised of a combination of several high and low levels. The low-level voltage was used to release the trapped charge in the dielectric layer. The high-level voltage was used to accelerate the activation of pixels, allowing the EWD to return to its original luminance prior to the application of the reset waveform. The insertion period of reset signal, the closing time $T_{off}$, the opening time $T_{on}$, and the maximum driving voltage $V_{max}$ in the reset driving waveform must be set in accordance with the oil backflow parameters in the EWD.

In the AM-EWDs, the TFT structure shown in Figure 1d is the key component for driving the pixels. The potential between pixel electrodes was indirectly regulated by the voltage on each pin of the TFT. As shown in Figure 6, the pixel voltage change caused by the voltage polarity flipping of the common electrode was analyzed when pixels were kept open. The initial state and three complete scanning frames are contained in the figure. Phase (a) represents the initial state when the electrode voltage between pixels was zero. Each scanning frame was comprised of two distinct phases. In the first scanning frame, the output voltage of the source electrode in phase (b) was −15 V. When the output of the gate electrode was high level, the actual voltage between the pixel electrodes was 30 V. When the polarity of common electrode voltage $V_{com}$ was flipped from positive to negative in phase (c), the TFT was in a state of open circuit due to the extremely high electrical impedance (about $8\times 10^{13}\Omega$). In an ideal state, the voltage between pixel electrodes remained at a constant level. One of electrodes in the pixel had a potential of −45 V, as illustrated by the red line segment. However, the source and the gate of the TFT would conduct to form a discharge circuit when the voltage difference $V_{gs}$ between the gate and the source was less than the threshold voltage $V_{th}$. The potential at one end of the pixel was reduced to −20 V by the limitation of gate voltage, as illustrated by the blue line segment. At this time, the actual driving voltage across the pixel was about 5 V. In the second scanning frame, the output of the gate electrode in phase (d) was a high level, and the actual driving voltage across the pixel was 30 V. When the voltage polarity of the common electrode was flipped during phase (e), $V_{gs}$ was still greater than $V_{th}$. Therefore, the TFT remained in a state of open circuit, and the actual driving voltage across the pixel remained at 30 V. Subsequently, the third and fourth frames would repeat the voltage changes of the first and second frames, respectively. With two scanning frames as a cycle period, the average driving voltage across pixels was 17.5 V. During this driving process, the voltage polarity between pixel electrodes did not change as the polarity of $V_{com}$ flipped. Accordingly, this methodology was only capable of generating the unipolar reset driving waveform illustrated in Figure 6 (S_5_).

In the experimental setup, the storage capacitor architecture of the AM-EWD was a $C_{s}$ on common structure. On the TFT substrate used in the experiment, a UC8430 chip was employed as the gate driver. The output of the gate driver was limited to high and low levels, which belong to a two-level addressing method. Nevertheless, one disadvantage of the two-level addressing method was that it generated a feed-through voltage that affected the voltage between pixel electrodes. In the principle of a two-level driving system, the generation of feed-through voltage was mainly due to changes in other voltages on the panel. The accuracy of the pixel electrode voltage was impacted by the feed-through voltage, which was transmitted through parasitic capacitance, storage capacitance, and pixel capacitance. Therefore, the influence of feed-through voltage on pixel voltage cannot be ignored during the driving process. When the common electrode voltage remained at a constant value, the most significant impact on the driving voltage of pixels was exerted by the feed-through voltage generated from changes of the gate driver voltage. At this point, the feed-through voltage $V_{ft}$ could be derived based on the conservation of electric charge law, as shown in Equation (1).

(1) $$V_{ft}=\frac{C_{gd}*(V_{g2}-V_{g1})}{C_{gd}+C_{pixel}+C_{s}}$$

where $V_{g1}$ and $V_{g2}$ represent voltages when the gate line is open and closed, respectively. $C_{gd}$, $C_{pixel}$, and $C_{s}$ represent the parasitic capacitance, pixel capacitance, and storage capacitance, respectively. Apart from the change of gate driver voltage, the driving voltage of the pixel was also influenced by the movement of oil under different voltages. The EWD panel used in experiments required a 30 V driving voltage to meet the driving demands. Given that the maximum output of the source driver was ±15 V, the common electrode voltage could only be set to either 15 V or −15 V. To eliminate the impact of feed-through voltage on the driving voltage of pixel electrodes, the voltage of the common electrode required adjustment. The compensation value of the common electrode voltage was calculated according to Equation (1). However, the pixel capacitance $C_{pixel}$ would change with the change of driving voltage during the driving process. Therefore, it was not possible to eliminate the feed-through voltage corresponding to each grayscale through a fixed compensation value of common voltage. The primary electrical parameters of the TFT substrate used in the experiment are shown in Table 2. It was found that the pixel capacitance increased from 0.14 pF to 0.45 pF when the driving voltage of the pixel increased from 0 V to 30 V. At a pixel capacitance of 0.15 pF, the pixel charging rate was 96%. When the pixel capacitance increased to 0.45 pF, the charging rate dropped to 94%. Therefore, the dynamic range of the pixel charging rate was between 94% and 96%. Calculations indicated that the feed-through voltage varied between 1.3 V and 1.6 V at this time. Consequently, the compensation voltage for the common electrode in practice required adjustment according to the pixel capacitance or the pixel driving voltage. To eliminate the impact of the maximum feed-through voltage, the compensation voltage for the common electrode was set to the maximum feed-through voltage.

The dielectric layer of the display device used in the experiment was a non-polar polymer Teflon with high electrical resistivity. There were some defects and impurities in the Teflon materials which could trap electrons or holes. When a voltage was applied to pixel electrodes, a local electric field was also generated on the surface of the Teflon film. The movement of free electrons or holes could be accelerated by the local electric field. This dynamic process increased the possibility of their interaction with defects or impurity structures in the material. As a result, the local electric field indirectly increased the probability of charge carriers (including free electrons and holes) being trapped by internal defects or impurities. Typically, a periodic reset driving waveform was employed to reduce charge trapping by the dielectric layer. It was equivalent to releasing the trapped charges by short-circuiting the upper and lower substrates. Charge trapping had an impact on the actual pixel driving voltage, which was reflected in the luminance variation through oil movement. Hence, the relationship between charge trapping and driving voltage could be investigated through luminance variations of the EWDs. It was assumed that the relationship among luminance L, driving time t, and driving voltage U is depicted in Equation (2).

(2) $$L(U,t)=\alpha U\left(t-T_{0}\right)+L_{0}$$

where α is the charge trapping characteristic parameter, representing the influencing factors of the EWD pixel structure and material. $L_{0}$ denotes the minimum luminance generated by oil’s light transmittance without a voltage applied on the EWD. $T_{0}$ indicates the starting time of oil backflow. It was assumed that luminance during oil backflow has a linear relationship with the driving voltage, as depicted in Equation (3).

(3) ∆L=β∆Q

where β is a constant coefficient. ∆L represents the luminance variation, and ∆Q represents the number of trapped charges. By combining Equations (2) and (3), the functional relationships among the applied positive and negative polarity voltage amplitudes and the luminance variation are shown in Equations (4) and (5).

(4) $$\alpha_{P}U_{P}\left(t_{P}{-T}_{0}\right)+L_{0}=∆L\left(U_{P},t_{P}\right)$$

(5) $$\alpha_{N}U_{N}\left(t_{N}{-T}_{0}\right)+L_{0}=∆L\left(U_{N},t_{N}\right)$$

where $U_{P}$ and $U_{N}$ represent driving voltages under positive and negative driving conditions, respectively. $\alpha_{P}$ and $\alpha_{N}$ are the charge trapping characteristic parameters under $U_{P}$ and $U_{N}$. $t_{P}$ and $t_{N}$ are the durations of $U_{P}$ and $U_{N}$, respectively. Since the number of trapped charges during the driving process should be maintained at zero, the relationship between the luminance variation during the positive and negative polarity driving processes is shown in Equation (6).

(6) $$∆L\left(U_{P},T_{P}\right)=∆L\left(U_{N},T_{N}\right)$$

By combining Equations (4)–(6), the relationship between the positive and negative driving voltages can be described by Equation (7).

(7) $$\frac{U_{P}}{U_{N}}=\frac{\alpha_{N}}{\alpha_{P}}\frac{t_{N}-T_{0}}{t_{P}{-T}_{0}}$$

Based on the above analysis, it was evident that the amplitude of the positive and negative polarity voltages could be determined by the driving time t and the charge trapping characteristic parameter α. The value of α can be approximately represented by the slope of the oil backflow curve. Assuming that the positive polarity driving voltage is 30 V, the corresponding driving duration is $T_{P}$. Similarly, for a negative polarity driving voltage of −10 V, the corresponding driving duration is $T_{N}$. If $T_{0}$ is taken as the starting time, then the relationship between the driving times $T_{P}$ and $T_{N}$ can be described by Equation (8).

(8) $$T_{P}=\frac{U_{N}}{U_{P}}\frac{\alpha_{N}}{\alpha_{P}}T_{N}$$

**Figure 7:**
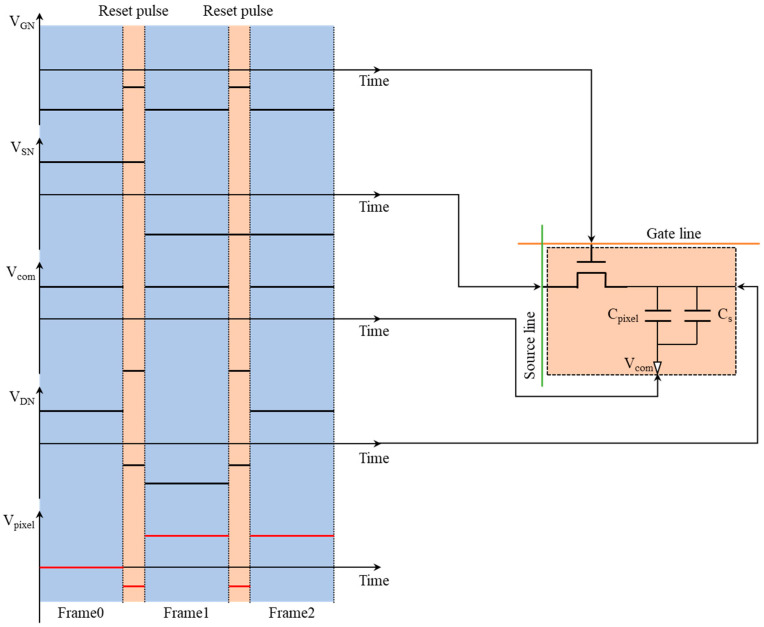
Schematic diagram of the inter-frame bipolar reset driving waveform. The TFT structure used to drive a single pixel is displayed in the right section. Within the electrical circuit, the EWD pixel was treated as a capacitive load. The voltage changes on various electrodes during the driving process are depicted in the left section. The driving voltage of pixels was determined by the output of gate, source, drain, and common electrode. The voltage variation on the TFT electrodes is shown as the black lines. The red lines represent the driving waveform applied between the pixel electrodes.

Based on the experimental results, $\alpha_{P}$ was found to be −0.0119 and $\alpha_{N}$ to be −0.7680. It was obtained that $T_{P}$ is equal to 21.5126 $T_{N}$ according to Equation (7). The driving time for the positive voltage of 30 V was 21.5126 milliseconds. The driving time for the reset voltage of −10 V was 1 millisecond. In AM driving, the duration of luminance fluctuation caused by the reset signal should be less than the duration of one frame. Building upon this insight, a driving approach with the frame-interleaved reset waveform was developed to minimize luminance fluctuation within frame duration. To meet the driving timing requirements of AM-EWD, the inter-frame reset waveform could only be applied during the vertical front porch (VFP) and horizontal back porch (HBP) periods. The pixel charge and discharge time on the TFT substrate was set as 33 microseconds. So, the refresh time for the active area of AM-EWD was 480 gate lines multiplied by 33 microseconds per line, totaling 15.84 milliseconds. At a refresh rate of 60 Hz, the available time for VFP and HBP was reduced to 830 microseconds. Consequently, the duration of reset driving waveform must be appropriately adjusted within 830 microseconds to meet the refresh time requirement of the active area. The schematic of the inter-frame bipolar reset waveform for the AM-EWD is illustrated in Figure 7. By adjusting the amplitude of the gate voltage $V_{Gn}$ during the reset period, the negative polarity voltage applied to the pixels could be regulated. The charge trapped by the dielectric layer during the continuous driving of the same voltage polarity could be release by the periodic switching of the voltage polarity between the pixel electrodes. In this driving method, the pixels in the display panel acted as the fundamental control unit. Therefore, the driving method was independent of the panel size and could be used to drive AM-EWDs panels of various sizes.

## 4. Experimental Results and Discussion

**Figure 8:**
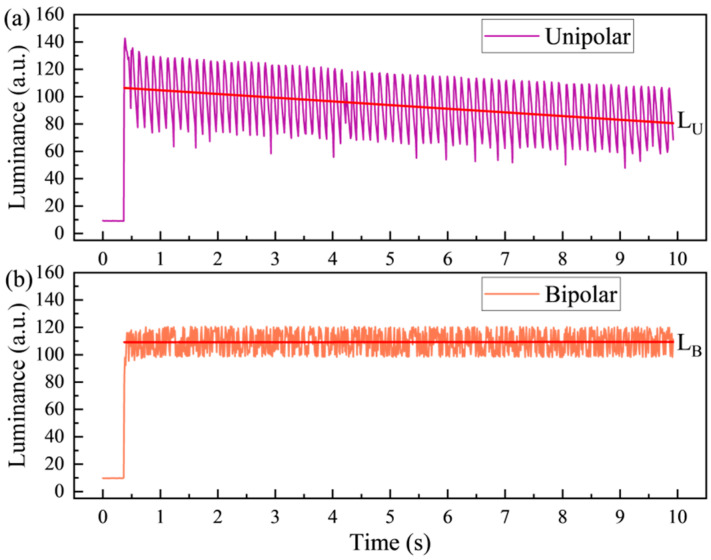
The luminance variation curve of the EWD panel under different driving waveforms. (**a**) The luminance curve of the EWD with the conventional unipolar reset waveform was applied [30]. (**b**) The luminance curve of the EWD panel with the bipolar reset waveform was applied.

To evaluate the influence of the bipolar reset waveform on the performance of the EWD, the luminance data from 0 to 10 s was collected by the colorimeter. The curves of EWD luminance under applied unipolar and bipolar reset waveform are shown in Figure 8. Because of the introduction of the inter-frame reset signal, the luminance curves of the EWD driven by both driving waveforms exhibited oscillations. With the same driving period, the standard deviation of luminance was 7.79827 a.u. for unipolar reset waveform and 19.04372 a.u. for unipolar reset waveform. It was indicated that the luminance of the screen exhibited a reduced fluctuation range when the EWD was driven by the bipolar driving waveform. In order to compare the effect of the two driving waveforms on the suppression of the oil backflow phenomenon, we performed a linear fit to the data after the luminance reached the maximum value. The fitting results are shown in Equations (9) and (10).

(9) $$L_{U}\left(t\right)=-2.70469*t+107.41124$$

(10) $$L_{B}\left(t\right)=-0.00262*t+109.10953$$

where $L_{U}\left(t\right)$ and $L_{B}\left(t\right)$ were the luminance fitting functions when a unipolar reset waveform and a bipolar reset waveform were applied, respectively. Based on the fitted linear function, the slope of the linear equation was −2.70469 a.u./s for the unipolar reset waveform and −0.00262 a.u./s for the bipolar reset waveform. It was indicated that the trapped charges could be released in time by applying the proposed bipolar reset waveform. Consequently, the EWD driven by the bipolar reset waveform was able to achieve a lower oil backflow rate and a more restricted range of the luminance fluctuation than the EWD driven by the unipolar waveform. In practice, the luminance of the display still exhibited a relatively slow decrease after the application of bipolar reset waveforms. It was observed that bipolar reset waveforms effectively suppressed the majority of reversible oil backflow. However, the inhibitory effect of bipolar reset waveform was quite limited when it came to irreversible oil backflow caused by defects or gaps in the dielectric layer. Consequently, the impact of the oil backflow phenomenon on EWDs was not completely eliminated.

**Figure 9:**
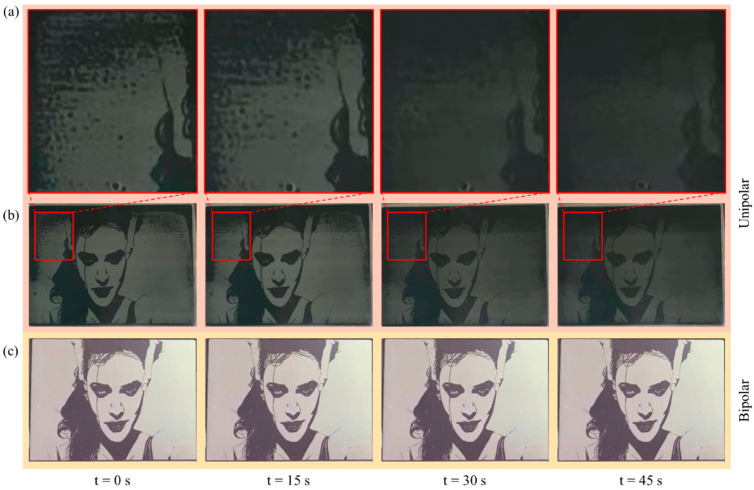
Comparison of EWDs display performance between bipolar driving and unipolar reset driving. (**a**) Partial enlarged view. (**b**) The display effective of EWD with unipolar driving. (**c**) The display effective of EWD with bipolar reset driving. A relatively high rate of oil backflow occurred in the areas within the red ellipse and rectangle in the image.

The enhancement of display performance resulting from the bipolar reset waveform could be visualized more clearly in the image. The display effect of a static image on the EWD through the application of unipolar and bipolar reset waveforms is illustrated in Figure 9.

As illustrated in Figure 9a, a relatively high-rate oil backflow occurred in the red rectangle region when the unipolar reset waveform was applied. The luminance of the entire screen also decreasing rapidly, as shown in Figure 9b. Especially in the red rectangle region, the ultimate pixel state was nearly in the “off” state. After a period of continuous driving, it was difficult to recognize finer details in the image. The display effect of the AM-EWD driven by the bipolar waveform is shown in Figure 9c. In contrast to the EWD with unipolar driving, the luminance of the screen was maintained in a stable state when the EWD panel was driven by the bipolar reset waveform. It indicated that the oil backflow in the AM-EWD could be effectively suppressed by the bipolar reset waveform. Consequently, the display stability of the AM-EWD was improved. The differences in display performance were caused by the varying efficiency of charge release between these two types of driving waveforms. The opportunity window for charge release in the unipolar reset drive waveform was limited to the zero-voltage driving phase. In the bipolar reset waveform, the accumulated trapped charges could be released by the opposite polarity driving voltage when the polarity of the driving voltage was changed. Furthermore, it has been demonstrated that non-zero-voltage driving exhibited superior charge release capability and speed in comparison to zero-voltage driving. It can be concluded that the application of a bipolar reset waveform in comparison to a unipolar reset driving waveform facilitates enhanced display performance in AM-EWDs.

## 5. Conclusions

In this paper, we investigated the bipolar driving principle in AM-EWDs with a storage capacitor on common. An appropriate compensation voltage for common electrodes was designed to eliminate the impact of feed-through voltage on the driving voltage of pixels. To meet the timing requirements of the TFT in AM-EWDs, an inter-frame bipolar reset driving waveform was designed. This waveform achieved a stable grayscale display by periodically releasing charges trapped in the dielectric layer during the driving process. Based on the designed experimental platform, we evaluated the display performance of a 6-inch display panel under both unipolar and bipolar reset driving waveforms. Compared to the conventional unipolar reset driving waveform, the decrease speed of luminance was reduced by 2.70 a.u./s, and the fluctuation range of luminance was reduced by 11.24 a.u. with the proposed inter-frame bipolar reset driving waveform. Therefore, the grayscale stability and image quality of AM-EWDs was enhanced by the proposed driving waveform.

## Figures and Tables

**Table 1:** Specifications of the EWD panel.

Parameters	Value	Unit
panel size	6	inch
resolution	648 × 480	px × px
width of pixel wall	12	μm
height of pixel wall	6	μm
thickness of dielectric layer	1	μm
thickness of ITO	25	μm

**Table 2:** Primary parameters of the TFT substrate.

Parameters	Symbol	Value	Unit
voltage of gate electrode	V_GH_	20	V
	V_GL_	−20	V
voltage of source electrode	V_SH_	15	V
	V_SL_	−15	V
voltage of common electrode	V_COM_	15	V
storage capacitance	C_s_	1.455	pF
pixel capacitance	C_pixel_	0.14–0.45	pF
parasitic capacitance	C_gd_	0.08	pF
	C_gs_	0.045	pF
	C_gp_	0.02	pF

## Data Availability

All data are contained within the article.
